# Innovative strategies for providing menstruation-supportive water, sanitation and hygiene (WASH) facilities: learning from refugee camps in Cox’s bazar, Bangladesh

**DOI:** 10.1186/s13031-021-00346-9

**Published:** 2021-02-26

**Authors:** Margaret L. Schmitt, Olivia R. Wood, David Clatworthy, Sabina Faiz Rashid, Marni Sommer

**Affiliations:** 1 Columbia University, Mailman School of Public Health, 722 W. 168th Street, New York, NY 10032 USA; 2 International Rescue Committee, 122 E 42nd St, New York, NY 10168 USA; 3 James P Grant School of Public Health, BRAC University, 68 Shahid Tajuddin Ahmed Sharani, Mohakhali, Dhaka, Bangladesh

## Abstract

**Background:**

There is growing attention to addressing the menstrual hygiene management (MHM) needs of the over 21 million displaced adolescent girls and women globally. Current approaches to MHM-related humanitarian programming often prioritize the provision of menstrual materials and information. However, a critical component of an MHM response includes the construction and maintenance of water, sanitation and hygiene (WASH) facilities, including more female-friendly toilets. This enables spaces for menstruating girls and women to change, dispose, wash and dry menstrual materials; all of which are integral tasks required for MHM. A global assessment identified a number of innovations focused on designing and implementing menstruation-supportive WASH facilities in the Rohingya refugee camps located in Cox’s Bazar (CXB), Bangladesh. These pilot efforts strove to include the use of more participatory methodologies in the process of developing the new MHM-supportive WASH approaches. This study aimed to capture new approaches and practical insights on innovating menstrual disposal, waste management and laundering in emergency contexts through the conduct of a qualitative assessment in CXB.

**Methods:**

The qualitative assessment was conducted in the Rohingya refugee camps in CXB in September of 2019 to capture new approaches and practical insights on innovating for menstrual disposal, waste management and laundering. This included Key Informant Interviews with 19 humanitarian response staff from the WASH and Protection sectors of a range of non-governmental organizations and UN agencies; Focus Group Discussions with 47 Rohingya adolescent girls and women; and direct observations of 8 WASH facilities (toilets, bathing, and laundering spaces).

**Results:**

Key findings included: one, the identification of new female-driven consultation methods aimed at improving female beneficiary involvement and buy-in during the design and construction phases; two, the design of new multi-purpose WASH facilities to increase female beneficiary usage; three, new menstrual waste disposal innovations being piloted in communal and institutional settings, with female users indicating at least initial acceptability; and four, novel strategies for engaging male beneficiaries in the design of female WASH facilities, including promoting dialogue to generate buy-in regarding the importance of these facilities and debate about their placement.

**Conclusions:**

Although the identified innovative participatory methodologies and design approaches are promising, the long term viability of the facilities, including plans to expand them, may be dependent on the continued engagement of girls and women, and the availability of resources.

## Background

Around the world, girls and women have distinct water, sanitation and hygiene (WASH) needs as compared to boys and males due to direct or indirect byproducts of their physiology, reproductive health processes, social and cultural norms and heightened vulnerability to violence [1]. Global evidence suggests that during humanitarian emergencies, girls and women experience increased challenges in relation to managing their WASH-related needs given the frequent lack of reliable access to safe, private and comfortable toilets and bathing facilities [2–4]. The management of monthly menstrual blood flow creates additional hurdles, as girls and women often have insufficient materials for catching menstrual blood, facilities for changing and disposing of menstrual waste, spaces and supplies for the laundering of reusable menstrual materials (e.g. pads, cloth or underwear) [4], and menstrual health and hygiene information [3, 5]. Privacy in such contexts is often non-existent, with toilets frequently lacking sufficient locks, doors, lighting and enforced gender segregation [6–9]. In many resource constrained settings, including emergencies, inadequate access to safe, clean and private toilets has been associated with increased experiences of stress [10, 11], embarrassment [6, 12, 13], physical discomfort, and gender-based violence [14–16].

In recent years, there has been growing interest in improving humanitarian response around menstrual hygiene management (MHM) [2, 3]. Current approaches often focus on the provision of menstrual materials [9, 17] and supportive supplies (e.g. buckets, drying lines, soap) [2, 7, 18, 19]. Such distributions usually include hygiene promotion (e.g. basic demonstrations on how to use distributed items) and menstrual hygiene or health-related education [18–20]. Reusable menstrual materials have also grown in popularity for distribution in emergencies [8, 18–21], as these are perceived to be more sustainable and cost effective, particularly in protracted emergency contexts. However, reusable materials require consistent access to soap or laundry detergent, along with private spaces for the discreet washing and drying of the materials; the latter of which is often lacking for girls and women in such contexts. Poor or inconsistent access to water for adequately cleaning themselves during menstruation, or for ease of washing reusable materials, is also often a challenge [13]. To date, there has been insufficient attention towards ensuring that WASH services are supportive of the use of reusable menstrual materials, reducing their potential effectiveness [2, 9].

The use of reusable and disposable menstrual material options necessitates a series of practical tasks that are not always physically or socially enabled in a humanitarian context. These include: one, the disposal of used menstrual materials, which includes both disposable and reusable types ultimately becoming waste; two, the washing and drying of reusable menstrual materials and underwear; and three, the safe and hygienic storage of menstrual materials when not being used or in between routine changings. Currently under-addressed in most responses, these tasks have direct implications for the design of WASH facilities. For example, the provision of covered drainage so that blood cannot be detected when flowing from a drain; providing water inside a cubicle to wash blood off hands or clothing; installing a waste bin or chute inside a toilet stall for discreet disposal of menstrual waste; or creating private laundering spaces for washing reusable materials (e.g. pads, cloths and underwear) that are hidden from view of other girls and women, men or children.

Menstrual disposal is also a complex issue to address in an emergency response given pervasive cultural beliefs and stigma around menstrual blood. In some contexts, girls and women have shared fears about witchcraft if used menstrual materials are seen by others [7, 22], or the risk of infertility or disease caused by the burning of menstrual waste [23–25]. In the absence of acceptable disposal options, girls and women often adopt coping strategies for discreetly getting rid of this waste, such as waking up before dawn to bury pads or cloth while it is still dark, which may pose safety concerns [6, 23, 26, 27] or disposing of used materials directly into toilets or latrine pits [5, 7, 23, 28]. There may be costly implications if menstrual waste is not considered from the onset of designing toilets, especially when large numbers of toilets are rapidly constructed. Menstrual waste can cause pipe clogging, faster pit filling rates, and difficulties in emptying toilet cesspits and septic tanks [6, 7, 23, 28, 29].

This paper describes findings that emerged from a project initiated in 2018 by the International Rescue Committee (IRC) and Columbia University’s Mailman School of Public Health (CU MSPH) aimed at expanding the evidence and guidance available on improving menstrual disposal, waste management and the laundering of reusable menstrual materials for girls and women in emergency contexts. This effort was undertaken subsequent to the publishing of the *Menstrual Hygiene Management (MHM) in Emergencies Toolkit* in 2017 [30]. The new global scoping exercise sought to fill evidence and practice gaps identified during the toolkit development process, and included a desk review and qualitative assessments conducted in three humanitarian contexts: Nigeria, Jordan, and Bangladesh. The aim was to identify new strategies being introduced for menstrual material disposal, waste management and laundering, and lessons about the design process, acceptability of approaches, and plans for maintenance over time. In this paper we describe findings specific to the assessment conducted in Cox’s Bazar (CXB), Bangladesh, given the breadth of innovative WASH approaches being introduced and piloted in that context.

## Methods

A qualitative assessment was conducted in the refugee camps located in CXB, Bangladesh to assess how the humanitarian response community, including non-governmental organizations (NGOs), United Nations (UN) agencies and other relevant actors, are addressing adolescent girls’ and women’s menstruation-related needs. This included capturing the perspectives of both beneficiaries and of emergency staff engaged in the response; including international and local (in-country) NGO personnel across a range of management levels.

### Study setting

Currently there are over 860,000 Rohingya refugees from Myanmar living in camps in southeastern Bangladesh [31]. These camps include basic shelters for housing, communal toilet facilities and a network of boreholes. Numerous NGOs are providing WASH programming, in addition to a range of other services including Health, Protection, non-food items (NFI’s), Shelter and Education.

Refugees are currently living in 34 camps in Ukhiya and Teknaf Upazilas [32]. Although critical improvements in services and access to water and sanitation have occurred since the acute phase in 2017, girls and women still experience significant challenges accessing toilets and bathing spaces, especially at night [4, 33]. A mix of disposable and reusable menstrual materials have been provided, with a growing shift towards the latter [4]. Recent assessments suggest that most girls and women prefer to either bury used menstrual materials or wash/reuse them, as was their practice before displacement [4]. Insufficient water access and spaces for drying menstrual materials have been identified as a challenge for many women in the camps and host communities [4]. Further, the location of the camps is prone to natural disasters, including flooding and landslides, conditions which can make the burial of menstrual waste problematic. The existing toilets were previously reported not to be female-friendly, as they often lacked gender-segregation and privacy, making them inaccessible to women due to safety concerns and cultural norms [4, 33].

In 2018, an inter-sector coordination group focused on MHM was developed by the WASH cluster in CXB which led to the development of a standards of practice guidance document [34]. Our assessment was conducted in consultation with the MHM Working Group leadership, and included capturing learning around MHM-related WASH response activities being piloted in select camps.

### Research design and methods

The objective of this assessment was to identify new approaches and strategies for addressing the MHM needs of girls and women in emergencies. Three types of data collection methods were used, including: 1) Focus Group Discussions (FGDs) with Rohingya adolescent girls and women; 2) Key Informant Interviews (KIIs) with NGO and Agency Staff, and; 3) Direct observations of WASH infrastructure.

### Sample and recruitment

The sample for the KIIs included a range of cross-sectoral humanitarian staff (male and female) from numerous organizations. Key informants (*n* = 19) were sampled purposively, with a focus on WASH and Protection actors involved in designing and implementing MHM programming. Thirteen of the KIIs were conducted in-person in CXB; 6 of the KIIs were conducted via Skype with international staff who had previously worked in CXB. The sample for the FGDs included adolescent girls and women between the ages of 15–49 years. Purposive sampling methods were utilized with the goal of including girls and women exposed to a variety of female-friendly WASH facilities, including new piloted innovations, in the camps. One FGD was conducted at a hospital serving both the refugee and host community. The FGDs were stratified into three age groups (15–18; 19–25; 26–49 years of age) in order to increase the comfort and participation of girls and women. A total of 2 FGDs per age group (*n* = 6) were conducted with a total of 47 participants. Each FGD was comprised of between 5 and 10 participants. The NGO partners in the camps facilitated the recruitment of adolescent girls and women.

The findings discussed in this paper are drawn from data collected across three different sources:

#### Key informant interviews (KIIs)

Interviews were conducted with NGO staff from different sectors (WASH, Protection), organizations and agencies to capture their perspectives on varying strategies for providing an MHM response in the CXB camps, the consultation practices utilized to solicit feedback from girls and women and new innovations in practice or design for MHM-supportive WASH facilities.

#### Focus group discussions (FGDs)

Semi-structured discussions were conducted with Rohingya adolescent girls and women, with questions examining girls and women’s experiences with managing their menstruation in the camps, challenges with the washing and drying of menstrual materials and their impressions of new WASH facilities.

#### Direct observations of WASH infrastructure

The research team also conducted a series of environmental observations of WASH facilities, which included taking photos and documenting various hardware and software design aspects.

Data collection occurred over a 2.5 week period in September 2019. The research team included 2 female staff from CU MSPH and 1 male staff from the IRC. All activities were conducted in confidential settings with a female translator who was trained on the importance of confidentiality. All FGDs were conducted in Rohingya with the 2 female research staff and the translator present. KIIs were conducted in English with all or some research staff (male and female) present. For the FGDs, tape-recording was not used to ensure the comfort of all participants. Instead, careful note-taking was conducted by the two team members, which included the capturing of both verbal and non-verbal responses. For the in-person KIIs in CXB, 12 of the interviews were recorded, and one handwritten due to participant preferences. All 6 of the Skype-based KII interviews were recorded. All participants provided oral informed consent prior to the start of data collection.

All study procedures were approved by the [blinded for review].

### Data analysis

Two members of the research team reviewed all qualitative transcripts (KII, FGDs) with the data analyzed using Malterud’s ‘systematic text condensation,’ a descriptive and explorative method for thematic analysis [35]. This approach utilizes a series of steps, including: a) broad impression, b) identification of the key themes, c) condensing the text from the code and exploring meaning, and d) synthesizing. Key themes identified were then shared with the larger research team for further validation, discussion and consensus.

## Results

Four key thematic areas emerged: 1) the utilization of female-driven participatory methods for WASH design; 2) shifts towards multi-purpose female WASH facilities; 3) the introduction of new menstrual disposal innovations at community and institutional levels; and 4) the use of male engagement strategies for generating buy-in for new female WASH facilities.

### Female-driven participatory methods for WASH design

Several organizations supporting WASH programs in the camps described utilizing a range of female- inclusive methods aimed to improve the solicitation process for female input and buy-in across the design and construction processes. Oxfam, for example, indicated the testing of a new iterative participatory approach, deemed “The Women’s Social Architecture Project.” With this approach, four female architects from BRAC University, in collaboration with Oxfam Hygiene Promotion staff, conducted a continuous series of discussions with groups of girls and women to solicit their opinions on the components of an ideal female-friendly WASH facility. This included specific groups comprised of older women, adolescent girls and females living with disabilities to ensure a range of perspectives were collected. A hygiene promotion staff member explained how they ensured it was a female-driven design process:

*… the technical people [WASH actors] didn't give any input. The engineers were just there to listen to what the community will tell. So, the women gave all the design ideas …*

Following initial consultations, a series of miniature models of WASH facilities were constructed from cardboard and brought back to the groups of females for further discussion and refinement. This included dialogue about the location for these facilities and what types of physical materials should be used for its construction. Program staff indicated that the location for the placement of the facilities proved to be a key concern, given the culturally restricted movement for most Rohingya girls and women in the camp. From conceptualization to construction, this process occurred over a series of 9 months.

**Fig. 1:**
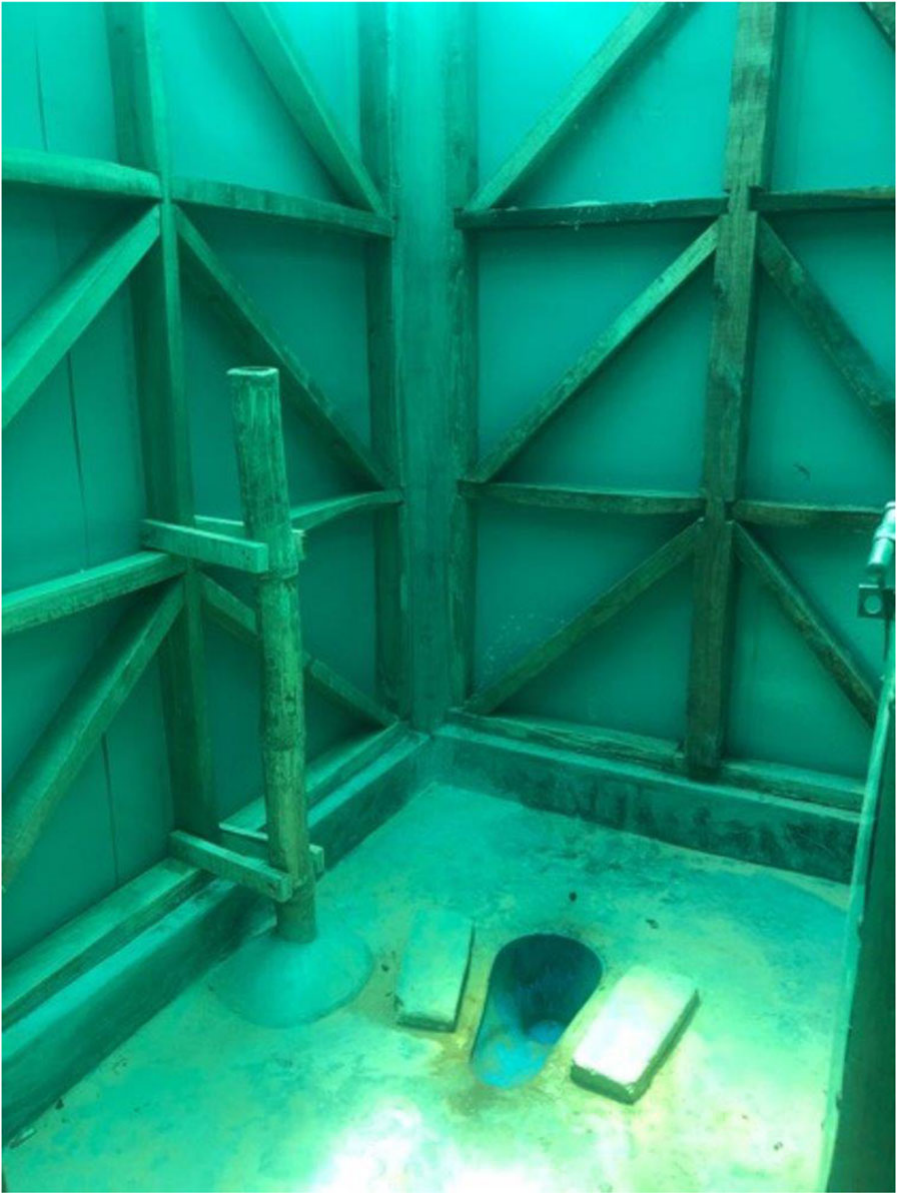
is an example of a toilet constructed by Oxfam which incorporates specific female-friendly features, including a bamboo pole to support pregnant and elderly users

During the social architecture process, females involved in consultations voiced a series of design concerns. This included fears that men and other community members could potentially peer inside the proposed new facility when girls and women were using it, especially given the hilly camp terrain. One hygiene promotion actor described how this expressed concern translated into a design approach in relation to determining the roof material as *“they didn’t want the transparent tin sheets often used because if the light comes through … others can see in. So it is now a green color.”* Beneficiary input was also described as informing the layout design and the introduction of new specific female-friendly elements, such as a bench built inside the bathing room, and a thick bamboo pole next to the latrine slab (see Fig. 1). As one older Rohingya woman explained the rationale for this design recommendation during the FGD:

*… pregnant women and elderly women, they have a support now to hold onto … you can sit and use it to steady [yourself] while you use water to clean up and then use it to get yourself up [from the latrine slab]...*

Other organizations, including the Bangladesh Red Crescent Society with support from the Danish Red Cross (BRCS/DRC), also tried to utilize female-driven participatory approaches when designing new sanitation facilities. However, they described encountering challenges when trying to recruit girls and women to initially participate in toilet design discussions. As one WASH advisor explained:

*… we could not even find women to participate during the meetings, and girls also. I asked why and they said “oh you know, in our culture … " but that's the reason why we wanted to develop it [new toilets] in the first place! Because we want to develop their voice and to hear about the problems they have with the latrines now...*

To address this challenge, the BRCS/DRC WASH actors coordinated with Protection colleagues to engage community volunteers in the effort to encourage participation by girls and women. The volunteers had personal relationships with many of the girls and women, and thus were able to convince them of the importance of sharing their preferences. Program staff indicated that over time girls and women became more comfortable vocalizing their thoughts about toilets and menstruation, and ultimately took on active roles as watch guards over the newly constructed toilets. The latter was an approach aimed at ensuring that the new toilets were properly maintained and cleaned. The improved dialogue with female users also enabled WASH actors to solicit their feedback regarding future design improvements or modifications needed. For example, a WASH actor described her surprised reaction after suggesting the introduction of lights inside the constructed toilets, as the Rohingya women immediately conveyed discomfort with this suggested modification. Their explanation was that “the men would know that we [females] are inside”; this in turn would make them feel unsafe. Other WASH actors further highlighted their commitments to continued involvement following construction, explaining:

*… even after handing it over, we still work on the facility. For example, so now they [female users] say to us: ‘we actually want a front door now.’ So we asked ‘what sort of front door?’ ‘covered or like a fence area … ?’ So then we make that one they want...*

Such improved consultation efforts, including after the design and construction phases, were critical for ensuring that girls and women felt comfortable using, and invested in maintaining, the new facilities.

### Shifts towards multi-purpose female WASH facilities

The improved female consultation processes also contributed to the eventual piloting of multi-purpose female-friendly WASH spaces. Several NGOs indicated that their consultations revealed girls’ and women’s indicated preferences for multi-purpose WASH spaces. This contrasted with the standard standalone blocks of toilets or washrooms constructed in most emergency contexts. The discussions revealed the increased privacy desires of beneficiaries, and on-going menstrual stigma experienced in the communities. As a Rohingya woman explained, a multi-function design would ensure *“*nobody would recognize whether I’m going to use the latrine, bathing space or for this [managing my menstruation] reason*.”* Such insights led the Oxfam team to construct a communal female-friendly WASH unit comprised of separate toilets, a shower stall and a laundering area, all within one structure.

A similar preference for combined facilities emerged from an assessment conducted with girls and women by CARE Bangladesh, which led the response team to integrate laundering facilities into an existing Protection Center. As girls and women were already routinely using the Protection space and considered it a safe, private and gender-segregated location, it seemed like an ideal location to pilot a multi-purpose design. As one NGO actor described the facility:

*… it created two layers of privacy. They have the privacy of the protection space, which in this context was incredibly important to women, with tall walls around it, but then also a separate laundering area inside the center … with internal privacy walls around that area as well.*

The laundry facility, which included a series of open laundry stalls lined up in a row with small platforms for sitting, also enabled girls and women to socialize while washing clothes. In order to provide them with an extra layer of privacy when washing more sensitive items, such as menstrual cloths or underwear, a shower curtain divider was placed between each stall. This divider, which could be opened or closed, enabled autonomy in deciding when additional privacy was desired.

Two main benefits emerged from the utilization of a multi-purpose space: the privacy it provided girls and women, and its convenience within their daily lives. As one NGO actor explained: “*…* no one would know if they were there to manage their menstrual laundry or day-to-day laundry … or to participate in other Center programming*.”* A third benefit described by the women utilizing the new space related to the presence of other community members and staff in the Center, who they could rely on for childcare while they completed laundry chores.

### The introduction of new menstrual disposal innovations at community and institutional levels

Adolescent girls, women and NGO staff all described menstrual product disposal as an ongoing challenge, especially given the absence of a comprehensive solid waste management system. This included how disposal practices were negatively impacting the functioning of WASH facilities. A number of NGO staff described how menstrual materials, including cloth, reusable pads and disposable products, were creating environmental issues, such as trash in roadside drains and the clogging of existing toilet facilities (e.g. pour flush models). As one WASH staff explained, “before [in the acute phase] it was a full disaster … in the latrines when we were doing desludging, the pumps were getting clogged because of the waste that was thrown into the latrine*.”* In the absence of acceptable disposal options, girls and women indicated that for privacy reasons they often preferred to bury menstrual waste, as burying was the practice they used prior to displacement. Further, they also feared that others, including men, children, other women and evil spirits might encounter their menstrual waste which was considered unclean and shameful. Burying waste however was proving increasingly difficult and problematic given the crowded conditions (e.g. closely packed household shelters) and flood prone nature of the camps. This in turn was perceived to impact girls’ and women’s mental health and wellbeing, as a WASH advisor explained:

*… they just want to be able to throw away the pads and not think about them … they’re worried when they bury them, they talk about how sometimes dogs dig them up and they’re so embarrassed, it’s so humiliating. Or if a man sees one of their pads, even an unused pad in a package, it’s really embarrassing. They have a lot of shame and bad feelings … there is a lot of common perceptions that women are unclean …*

**Fig. 2:**
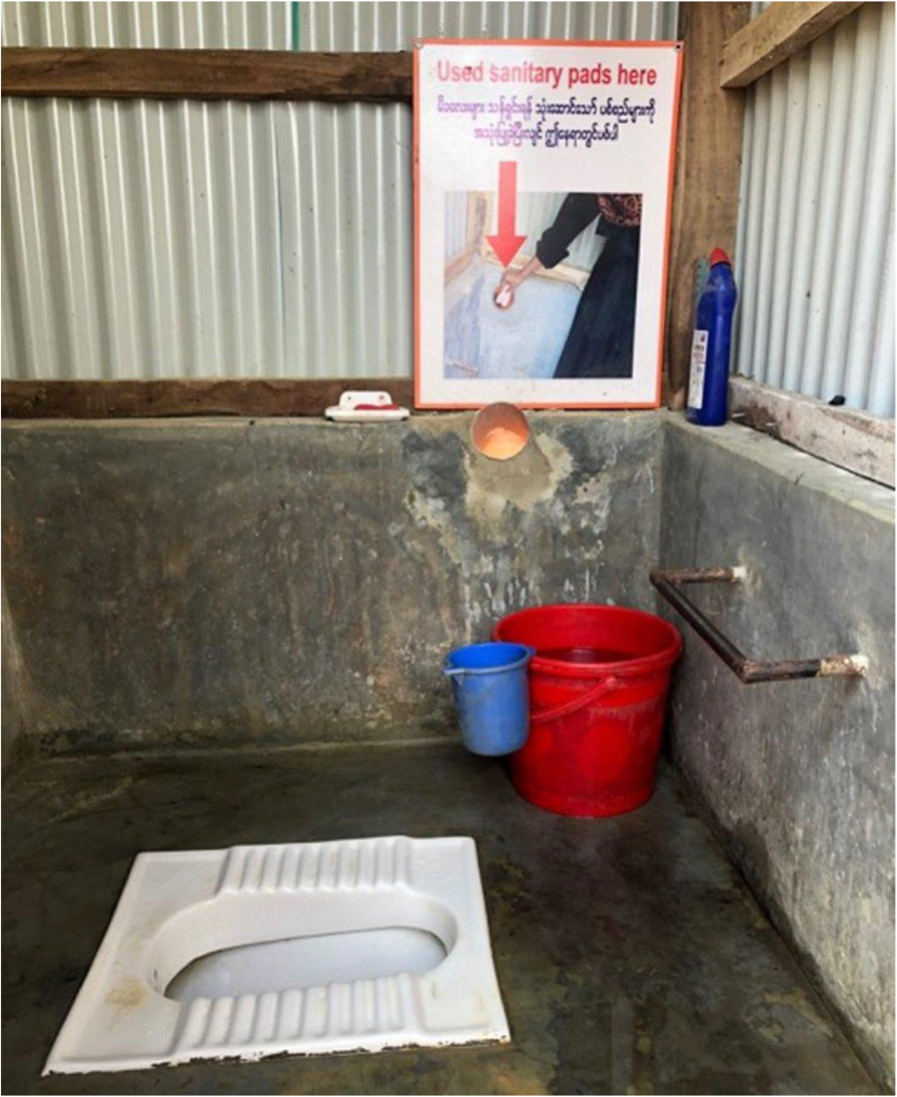
is an example of a toilet constructed by BRCS/DRC, which includes female-friendly features like a metal bar to support pregnant and elderly users, water directly inside the latrine stall and a menstrual waste chute, with appropriate signage, for the disposal of used menstrual products

**Fig. 3:**
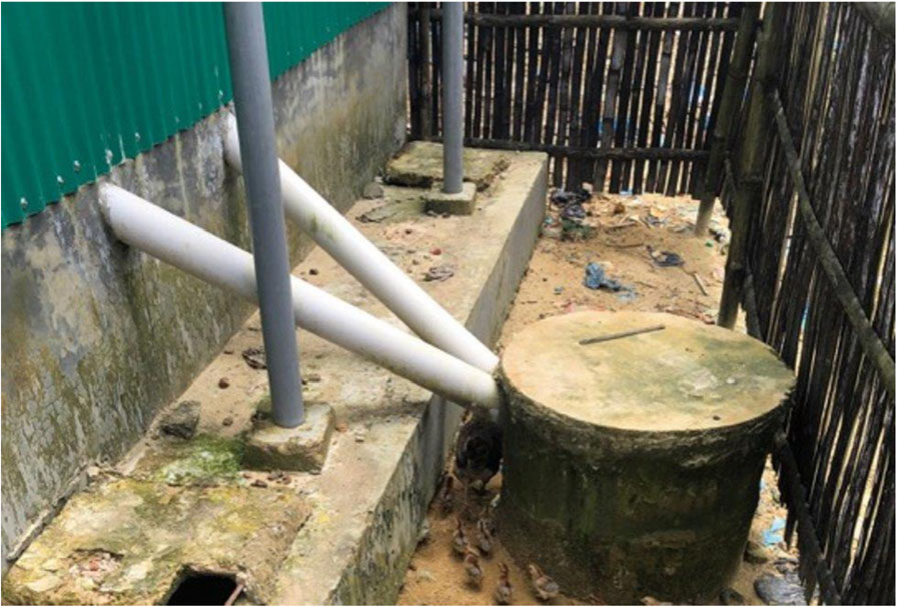
displays external containers outside the BRSC/DRC female latrines where menstrual waste inserted into the disposal chutes is stored

To address these challenges, a few NGOs designed or are piloting hardware and software solutions aimed at improving the handling of menstrual waste so that it would no longer be dropped into toilets or buried outside. For example, BRC/DRC introduced a menstrual disposal design as a component of their more female friendly toilets that incorporated a discreet chute system for transferring menstrual waste from the inside of the toilet cubicle into a protected cement container external to the toilet (see Fig. 2). A polyvinyl chloride (PVC) pipe chute was inserted into the back wall of the toilet to transfer this waste. The external storage container cannot be readily accessed or viewed by users, a design feature particularly important to girls and women (see Fig. 3). One Rohingya woman explained how the new disposal chutes helped to address the cultural beliefs and taboo surrounding menstrual product disposal:

*… we have worries about if blood is seen by others or if evil spirits may find it, so now we know it is safe from this. We can throw it away and put it in that system [the chute] and we do not worry about it. We are free from worries about the spirits now …*

To ensure that all girls and women understood how to use the new disposal option, community sensitization sessions and demonstrations were conducted, and signage was developed using visual depictions to promote proper usage. The latter was particularly important given literacy concerns amongst this population. Although these new toilets had been installed for less than a year, girls and women expressed initial enthusiasm for the disposal design, viewing it as an acceptable, and even desirable, alternative for disposing of menstrual waste. As another Rohingya woman described, *“*we like it [the chutes] because we don’t have to bury the cloths anymore. We don’t have to dig a hole and it is not seen by the children … it is not seen by the males. It is secure for us whenever I want to use it*.”*

**Fig. 4:**
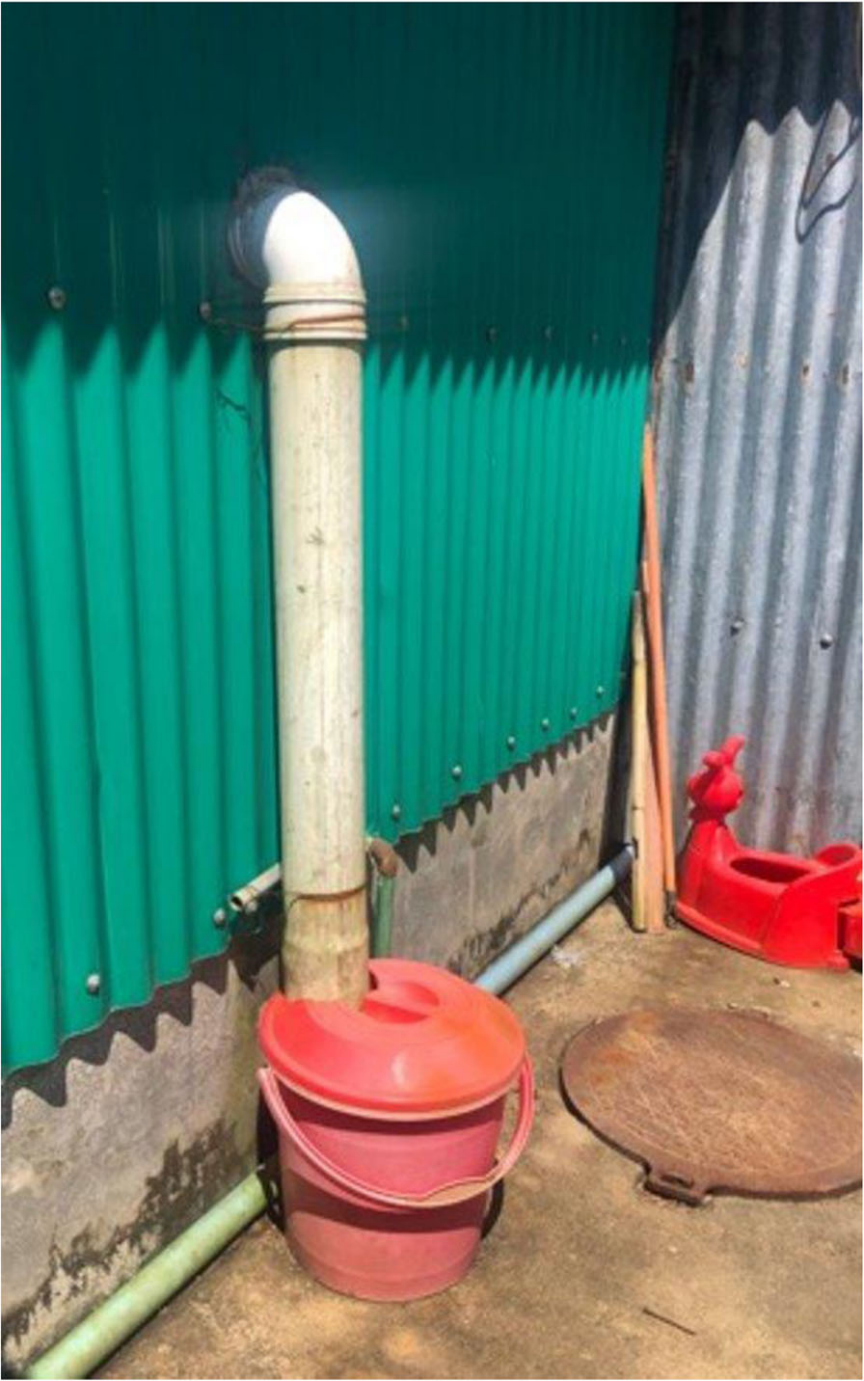
displays external containers outside the MSF hospital female latrines where menstrual waste inserted into the disposal chutes is stored until onsite incineration

In terms of institutional WASH approaches, a new menstrual waste design was identified at a hospital facility operated by Medicines San Frontiers (MSF). This facility provides in-patient healthcare to refugees and the host community. Hospital staff distributed disposable menstrual pads to both menstruating and post-partum female patients and their caregivers. Two approaches were developed for menstrual waste within the toilet stalls of the facility: one, the use of pedal-operated color-coded (red) bins, and, two, the use of a disposal chute system in female toilets and showers. The latter used a PVC pipe directly inserted into the back wall of the toilet or shower structure, leading to an external covered plastic red bin located behind the toilet and bathing facilities (see Fig. 4). The red bins were part of a larger color-coded waste management system which enabled sanitation staff to more easily sort waste streams for final disposal at an on-site incinerator. Although the extent to which the disposal chute system was utilized by beneficiaries could not be readily determined (as the research team did not have an opportunity to interview direct users), girls and women did indicate the acceptability of using the pedal-operated red bins. One reason was their appreciation for the frequent emptying of bins by hospital cleaners supplied with protective gear, which diminished the potential for others to see their menstrual waste. Both approaches enabled more discreet disposal for the patients and created more hygienic solutions for handling menstrual waste in a medical setting.

### Use of male engagement strategies for generating buy-in for new female WASH facilities

Several NGOs made significant efforts to engage men in the design of facilities for girls and women. This emerged as an important aspect of the WASH design process for improved MHM, although it may also have been particularly relevant in the Rohingya camp context where men to a large extent control girls’ and women’s movement outside the household. An example of one effort included BRC/DRC staff engaging men from the beginning when they explored how best to include menstrual disposal in new female toilets. Hygiene promotion staff initiated discussions with Rohingya male volunteers who were involved in the community-led toilet construction efforts, positing to them: *“*Do you even know where your women throw their menstrual pads*?”* This led to a dialogue on the challenges females face in their community when disposing of menstrual waste, along with underscoring how men need to be part of the solution. Rohingya men subsequently supported staff with designing the new disposal hardware, and with constructing the female toilets with the new features. Men also indicated a willingness to serve a role in the waste management system, agreeing to be responsible for emptying the concrete drums for incineration every 3 or 4 months for their shared household latrines. The success of the emptying component has not yet been determined as not enough time has passed since their introduction.

Similarly, Oxfam sought to generate male support about the importance of creating distinct WASH spaces for women and girls. There was a strong sense that given the sociocultural norms of the Rohingya community, such an approach would increase the future usage of the latrines. As one Hygiene Promotion actor explained:

*… we talked to the male persons regarding this new model … we collected 20 men to talk to. So we say...“women want this this thing and this thing in a [WASH] facility … so what do you think?” Some had opinions, but later on after more discussion everyone agreed upon this thing … that yes, this will be good for the women...*

As noted already, a cultural aspect of great relevance to the design process of the facilities was the limited mobility of Rohingya girls and women, who primarily remain near their homes, only leaving with the permission of male household leaders. This restricted movement created challenges for bathing, accessing toilets and managing menstruation, especially during the night time. As one WASH actor explained:

*… some WASH facilities are beside the road and they have no privacy protection, and as this is a Muslim ethnic group … girls are not feeling free to move in the daylight. Also, you know people cannot hold … [their sanitation needs] for a long time. So where are they going? Inside their home …*

The WASH staff also engaged with men around the placement of the latrines out of concern that if girls and women were hindered from accessing toilets due to their location, beneficiaries might construct makeshift household toilets and showers, creating hygiene concerns. The engagement process with men included significant debate about latrine locations, with one hygiene promotion staff member describing how:

… *every male person was saying that if the facility is nearby my house, I will let my woman or girl go use that one. If it is just a bit far, then maybe I will not let my ladies go use the washroom …*

However, through continued discussions, the team worked to identify compromises and eventual buy-in by many male participants. A key learning was the importance of engaging the future users of the latrines in such efforts, which proved complicated given space limitations for facilities in the camps.

## Discussion

The findings from this qualitative assessment conducted in the Rohingya refugee camps in CXB highlight increased efforts by NGOs to more inclusively engage girls, women and men in the design process for improved WASH facilities that are more supportive of MHM in a humanitarian context. This includes utilizing new forms of participatory approaches for soliciting sensitive information across the design phases. Findings revealed that emergency actors are now looking beyond the distribution of menstrual products and examining what else girls and women need to more effectively address their sanitation needs in relation to and beyond menstruation. This includes the design of more female-friendly toilets, and also user-adapted bathing and laundering spaces [36, 37]; all components that are vital for a holistic MHM response [7].

A particularly effective approach that emerged from the MHM programming in CXB was ensuring female voices had a larger role in the design process. The use of “feminist design” [37] has been found in other emergency contexts [38, 39], especially by those developing adolescent girl focused programming [40, 41]. Similar strategies have been incorporated into girls’ and women’s programming across a range of development contexts [42–45]. A crucial element, which this limited assessment was not able to fully examine in the context CXB, is the need to engage beyond the design phase. This includes determining strategies to sustain female engagement over time, for example, to assure toilet facilities are utilized and maintained as planned. This is particularly critical given the protracted nature of so many humanitarian contexts. Furthermore, such objectives align with broader humanitarian principles which call for programs that aim to increase community ownership, control and decision-making [46]. Even a toilet built for and accessible to only one family may retain some ambiguity around maintenance and cleaning if the family sees the toilet as the property of the NGO that built it. Such ownership and maintenance issues may be more complex for toilet facilities shared by several families, or for those which are public facilities [47]. Routine consultation with female users during on-going programming can help to ensure that facilities continue to operate as intended. This engagement, including mechanisms for feedback, serves to sustain female buy-in over time, which may enhance the facility’s operational capacity and improve community-led oversight and trouble-shooting. In Cox’s Bazar, community members were responsible for the cleaning and maintenance and some of the operations of the communal latrines shared by households; these proved to be effective approaches.

As organizations explore new methodologies for consulting girls and women in humanitarian settings, such approaches should be adapted to the sociocultural context in which they are operating. Many organizations and researchers are beginning to move beyond traditional one-time focus group discussion (FGDs) or short interviews, and they are exploring strategies which involve multiple rounds of discussions. These strategies are more participatory and/or have opportunities for refinements before determining a path forward [37, 38, 41, 48]. Such iterative approaches to WASH practice, although requiring more time and capacity, may result in the development of more responsive solutions to the needs of girls and women. This in turn may translate into increased facility use and improved health and wellbeing. Previous research also indicates the dangers of poorly designed and maintained sanitation facilities for girls and women. For example, an inappropriate placement of toilets (proximity to male-designated toilets) or lack of door locks can enhance the potential for male intruders or “peeping toms” thus putting girls and women at heightened risk for gender-based violence [38]. Although many of these iterative design processes are being tested in protracted emergency contexts [18, 37, 38], important WASH practice lessons can be derived from these strategies for potential use in more acute responses requiring rapid design. This includes the importance of having participants visualize and reflect on conceptualized facilities or programming, including how they will interact with the design, prior to construction [37, 49]. Such approaches may prove valuable, and even cost effective [38], in terms of lifetime usage and uptake.

Of particular importance, the emergence of multi-purpose WASH facilities was a direct by-product of improved consultation methods with girls and women. A key finding from this study was their voiced emphasis on the benefits to be gained from multi-purpose WASH spaces for promoting a sense of privacy and reducing shame. This is underscored by the literature highlighting the discomfort females experience accessing toilets in many cultures and contexts [10, 13, 50]. The provision of multi-purpose WASH spaces may yield additional benefits, such as enhancing opportunities for socialization and social support systems among girls and women. This can be especially important in emergencies where female mobility may be limited, or family support structures diminished. These spaces can also encourage collaborative child care arrangements, enabling girls and women to accomplish hygiene and household-related tasks while visiting the space. Combining facilities may also be strategic from a design perspective, as both bathing and laundry spaces are dependent on access to a water point [37]. Lastly, multi-purpose WASH spaces may help to diminish the stigma girls and women feel when having to change, wash or dispose of their menstrual materials in facilities. Many organizations in both development and emergency contexts have attempted to address girls and women’s menstrual needs by creating menstruation-specific rooms or toilets stalls, often equipped with water, drains or drying lines. However, these menstruation-specific rooms are often met with resistance from girls and women given the high levels of stigma associated with their usage [7]. Multi-purpose WASH spaces may mitigate this stigma by prioritizing girls and women’s privacy when managing menstruation, be it related to bathing, laundering, or product changing and disposal. More research is needed, however, that examines the acceptability and cost-effectiveness of introducing multi-purpose WASH facility approaches, including across varying contexts.

New innovative approaches for menstrual product disposal in humanitarian contexts, including the types of consultative processes used in the design phase, are a particularly critical contribution to the field that emerged. This includes a concerted effort by NGOs to consider the strong influences of menstrual-related stigma and taboos, particularly around menstrual waste, when devising solutions. Although the programming described here is very promising, particularly given the positive initial user feedback, there is an urgent need for operational research to ensure a sustained viability and acceptability of the designed approaches. Approaches utilized in development contexts suggest challenges can emerge around the maintenance of new disposal systems, such as volunteer-led cleaning systems being difficult to sustain over time [51]. There is a need to test not only new hardware solutions (e.g. disposal chutes) and related software approaches promoting their usage, but also the corresponding waste management systems needed to ensure that a design such as a chute continues to operate effectively. This may include, for example, exploring how best to train cleaners and waste management staff on handling menstrual waste, so as not to increase the stigma experienced by female users [6]. Lastly, there still remains a lack of consensus globally on how best to manage the final disposal of menstrual waste in many development and emergency settings [28]. As humanitarian responders continue to expand their distributions of menstrual materials to displaced girls and women, considerations for final disposal solutions remain a critical and often overlooked component of an MHM response.

### Limitations

There are some limitations to note. First, this research was conducted during a 2.5 week window. Given the limited timeframe, the researchers were unable to observe how beneficiaries interacted with these new WASH facilities over time, including whether any operational or acceptability challenges emerged and if female-engagement strategies persisted. Second, given that many of these new WASH innovations were relatively new and implemented on a limited or pilot scale, only a portion of the FGD participants interviewed had been sufficiently exposed to the new WASH facilities. Third, as the researchers were dependent on Rohingya translators for communication with beneficiaries, it is possible that there have been some miscommunications or deviations from the original messaging during translation of the FGDs.

## Conclusion

This scoping assessment of improved WASH facilities for supportive MHM in refugee camps in Bangladesh highlighted the importance of humanitarian responders using more iterative and participatory approaches for consulting with girls and women across the design and implementation phases, and the resulting benefits of implementing more user-centered facilities, including enhancing usage and the health and wellbeing of female beneficiaries. Sustained community involvement with both males and females was identified as essential for the implementation of a holistic MHM response. The latter includes attention to the full spectrum of girls’ and women’s menstruation-related needs, ranging from menstrual waste disposal to the laundering of reusable materials. Innovation in the design of WASH facilities, including the use of multi-purpose spaces and discreet disposal mechanisms, are critical for pushing the field forward, and importantly prioritizing girls’ and women’s sanitation privacy. The success and ultimate sustainability of any new MHM programming or facility design is likely to be dependent on how carefully responders consult with girls, women and their communities over time. Abbreviations:

## Data Availability

The dataset generated during this assessment are not publicly available due to the highly personal nature and detailed description of the very personal experiences in relation to menstruation and personal hygiene and sanitation which formed the basis of the qualitative interview and focus group guides. Furthermore, during the informed consent process, participants did not consent to making the data publicly available. Nonetheless, de-identified data may be made available by the corresponding author on reasonable request.

## Abbreviations

BRC: Bangladesh Red Crescent Society
CU MSPH: Columbia University’s Mailman School of Public Health
CXB: Cox’s Bazar
DRC: Danish Red Cross
FGD: Focus Group Discussion
IRC: International Rescue Committee
KII: Key Informant Interview
MHM: Menstrual Hygiene Management
MSF: Médecins Sans Frontières
NFI: Non-food Item
NGO: Non-governmental Organization
PVC: Polyvinyl Chloride
UN: United Nations
WASH: Water, Sanitation and Hygiene
